# The role of NaHS pretreatment in improving salt stress resistance in foxtail millet seedlings: physiological and molecular mechanisms

**DOI:** 10.1080/15592324.2023.2276611

**Published:** 2023-11-02

**Authors:** Xiao Zhang, Yuqin Ding, Miao Yang, Aili Wei, Dongao Huo

**Affiliations:** College of Biological Sciences and Technology, Taiyuan Normal University, Jinzhong City, Shanxi Province, China

**Keywords:** Salt stress, H_2_S, seedling growth, antioxidant enzyme activity, gene expression

## Abstract

Salt stress is a prevailing abiotic stress in nature, with soil salinization becoming a pressing issue worldwide. High soil salinity severely hampers plant growth and leads to reduced crop yields. Hydrogen sulfide (H_2_S), a gas signal molecule, is known to be synthesized in plants exposed to abiotic stress, contributing to enhanced plant stress resistance. To investigate the impact of sodium hydrosulfide hydrate (NaHS, a H_2_S donor) on millet’s response to salt stress, millet seedlings were subjected to pretreatment with 200 μM NaHS, followed by 100 mM NaCl stress under soil culture conditions. The growth, osmotic adjustment substances, antioxidant characteristics, membrane damage, and expression levels of related genes in millet seedlings were detected and analyzed. The results showed that NaHS pretreatment alleviated the inhibition of salt stress on the growth of foxtail millet seedlings, increased the proline content and antioxidant enzyme activities, as well as the expression levels of *SiASR4*, *SiRPLK35* and *SiHAK23* genes under salt stress. These findings demonstrated that NaHS pretreatment can enhance salt tolerance in foxtail millet seedlings by regulating the content of osmotic adjustment substances and antioxidant enzyme activity, reducing electrolyte permeability, and activating the expression of salt-resistant genes.

## 1. Introduction

Millet [*Setaria italica* (L.) Beauv.] is an annual herb plant widely planted in the temperate and tropical regions of Eurasia, with the upper and middle reaches of the Yellow River in China being the main cultivation areas. It is a typical environment-friendly crop with excellent characteristics such as drought resistance, salt tolerance, high water use efficiency, wide adaptability, tolerance to poor soil conditions, and strong yield stability.^1,2^ Due to its rich nutritional and health values,^3,4^ as well as the increasing demand for diversified dietary structures in contemporary society, there is a growing demand for foxtail millet. However, under the environment that crop yield and quality are affected by salt damage in China, this demand currently faces both opportunities and challenges.^5,6^

Soil salinization and alkalization has become an increasingly serious global problem. According to authoritative statistics, approximately 9% to 34% of the global irrigated land has suffered from the adverse effects of salinization to varying degrees.^7,8^ In China, the area of saline-alkali soil exceeds 99.13 million hm^2^, accounting for 4.88% of the total available land.^1^ Due to rapid population growth, improper irrigation practices, unreasonable use of fertilizers and pesticides, as well as global climate change, the rate of soil salinization has far exceeded expectations.^9^ Saline-alkali soils, rich in chloride and sulfate compounds, which cause salt stress on plants and severely affect their growth and development. Consequently, this leads to low seed germination rates, impaired antioxidant system, disrupted plant biochemical and molecular mechanisms, and ultimately resulting in a significant reduction in yield.^10,11^ As a result, grain production in China is severely restricted, impeding sustainable agricultural development.^12^

Research has revealed that different plant species possess the ability to mitigate the detrimental effects of salt stress on crucial plant aspects such as osmotic adjustment substances, membrane permeability, chlorophyll levels, and antioxidant enzymes. This is achieved through regulating their own metabolites or exogenous substances.^13–16^ Therefore, it is of great significance to further study the pathways and mechanisms of millet seedlings in response to salt stress and other signals.^17^

In recent years, one popular method to alleviate salt stress is the application of exogenous substances.^18^ As a gas signal molecule,^19^ H_2_S plays an important role in plant growth and development as well as response to abiotic stresses.^20^ Because H_2_S and NaHS can form a dynamic equilibrium in plants, NaHS is often used as a donor of H_2_S in biological research.^19^ Since 1990s, studies have shown that NaHS, as an exogenous regulator, can participate in the process of plant resistance to salt, cold and hypoxia. NaHS can alleviate oxidative stress in the cells by inhibiting the activity of glyceride oxidase in *Arabidopsis thaliana*. Moreover, it effectively modulates the accumulation of osmotic adjustment substances in cucumber seedlings under salt stress. In addition, NaHS can reduce the damage caused by salt stress through regulating antioxidant enzymes such as superoxide dismutase (SOD), peroxidase (POD) and catalase (CAT) in rice seedlings^21^ and improve the tolerance of millet seedlings to chromium by synergizing with Ca^2+^.^22–24^ Therefore, investigating the potential of the gaseous signaling molecule H_2_S to alleviate salt stress in millet and understanding its underlying mitigation mechanisms holds significant guidance for enhancing millet yield and economic benefits in the future.

Abscisic acid-, stress-, and ripening (ASR)-induced proteins are a class of proteins widely found in higher plants, and are induced by many abiotic stresses such as early drying, high salt, low temperature, H_2_O_2_, and ABA.^25^ The transcription level and protein level of tomato ASR1 were both up-regulated under drought and salt stress, and the transgenic tobacco heterologously expressing tomato ASR1 showed stronger drought tolerance and salt tolerance.^26,27^ The KT/HAK/KUP (HAK) family is the most abundant potassium transporter family in plants and plays an important role in plant growth and environmental adaptation.^28,29^ Lectin receptor-like protein kinases (LecRLKs) are the largest gene family in plants and play an important role in regulating plant growth and development, stress and disease resistance.^26^ A large number of studies have shown that LecRLKs expression is significantly up-regulated under salt stress conditions, which can reduce the accumulation of ROS to improve the salt tolerance of plants.^30^ In previous studies, the expression of these genes was upregulated in millet under salt stress,^31,32^ however, the effect of NaHS pretreatment on the expression of these genes in millet seedlings under salt stress is still unknown.

In order to study the effects of exogenous H_2_S on the growth and physiology of millet seedlings under salt stress, the growth, membrane damage, antioxidant capacity and osmotic adjustment substances of millet seedlings under salt stress were determined. The aim of this study was to reveal the physiological and molecular mechanisms of exogenous H_2_S in improving salt tolerance of millet and provide theoretical basis for breeding salt-tolerant millet germplasm, cultivating millet in saline-alkali soil and improving salt tolerance of millet.

## 2. Materials and methods

### 2.1. Plant material and plant treatment

In this study, ‘Jingu 21’ was used as the experimental material. The seeds of Jingu 21 were provided by Shanxi Agricultural University.

Before the experiment, seeds with full and consistent particles were selected and disinfected with 75% ethanol solution for 1 min, rinsed with ultra-pure water for 3 times, soaked in darkness for 24 h under 25°C, seeds were evenly seeded in a tray containing seedling substrate, with 25–30 seeds in each hole. After seeding, seeds were placed in an incubator with 25°C, 60% relative humidity and darkness for 48 h. After the seed germ was exposed, the incubator was set at 16 h light/25°C and 8 h darkness/23°C for normal growth. When the seedlings have grown to two leaves, the seedlings can be treated, the NaHS solution prepared with ultra-pure water is sprayed on the leaves until the solution is fully hung on the leaves, once in the morning and once in the evening, and treated for three days. NaCl solution was prepared with ultra-pure water and the seedlings were subjected to salt stress by watering. The control group was watered with ultra-pure water and samples were taken for testing of various indexes. After 7 days of treatment, leaf samples of different treatments were collected, some fresh samples were used for plant height and fresh weight determination, some fresh samples were used for phenotypic analysis (10 millet seedlings per treatment were taken for three biological replicates for phenotypic analysis), and some samples were rapidly frozen with liquid nitrogen (frozen samples were stored in a −80° refrigerator) for physiological determination and gene expression analysis.

### 2.2. Determination of endogenous H_2_S

After different treatments, 0.2 g millet seedling leaves were ground with 2 ml 50 mmol/L PBS (containing 0.1 mol/L EDTA and 0.2 mol/L ascorbic acid, pH 6.8), centrifuged at 10,000 g for 10 min at 4°C. Put the supernatant into a small triangular flask, put 1.5 mL EP tube added with 500 μl of 1% ZnAc into the small triangular flask, and then add 1 ml of 1 mol/L HCI to start the reaction: quickly seal the small triangular flask, place it at room temperature for 30 min, take out the EP tube in the small triangular flask, add 200 μL of 20 mmol NN-p-dimethyl-phenylenediamine and 200 μl of 30 mmol FeCl, shake it up, place it in the dark at room temperature for 15 min, and determine the absorbance value at 667 nm wavelength. Standard curve made with NaHS of different concentration.^33^

### 2.3. Determination of soluble sugar content

Soluble sugar was determined by Bates method.^34^ Weigh 1 g of anthrone and dissolve it in 1000 mL of dilute sulfuric acid (prepare by diluting 760 mL of concentrated sulfuric acid with distilled water to a volume of 1000 mL, with a relative density of 1.84), Transfer the solution to a brown bottle and keep it for immediate use. Plant leaves were oven dried at 110°C for 5 min and then adjusted to 70°C overnight. Dry leaves were ground into powder, 50 mg of the sample were poured it into a 10 mL graduated centrifuge tube, add 4 mL of 80% ethanol, place it in a water bath at 80°C and stir continuously for 40 min, centrifuge, collect the supernatant, add 2 mL of 80% ethanol to the residue and extract twice, and combine the supernatant. Add 10 mg of activated carbon to the supernatant, decolorize at 80°C for 30 min, dilute to 10 mL with 80% ethanol, and take the filtrate for determination after filtration. The sugar content in the extract was determined by the standard curve.

### 2.4. Determination of proline content

Proline content was determined according to the method of Shams.^35^ Weigh and cut 0.2 g of fresh millet leaves, add 5 mL of 3% sulfosalicylic acid solution, extract in boiling water bath for 10 min, after cooling, absorb 2 mL of supernatant, add 2 mL of glacial acetic acid and 2 mL of 2.5% acidic ninhydrin color developing solution, react in boiling water bath for 1 h, after cooling, add 4 mL of toluene solution, fully oscillate to extract red substance, stand still for stratification, absorb the upper toluene layer, compare the color under wavelength 520 nm, check the proline content corresponding to the standard curve, and calculate the proline content in millet leaves.

### 2.5. Determination of antioxidant enzyme activity

Accurately weigh 1 g of leaves to be tested and put them into a mortar, add 5 mL of precooled phosphate buffer solution with pH 7.8, grind them into homogenate in ice bath, transfer them into a 10 mL centrifuge tube, centrifuge at 12,000 r/min^−1^ for 20 min at 4°C, and collect the supernatant as the crude extract of enzyme. Photochemical reduction method of nitrogen blue tetrazolium is used to determine SOD,^35^ add 1.5 mL phosphate buffer solution, 0.3 mL methionine solution, 0.3 mL NBT nitroblue tetrazolium solution, 0.3 mL EDTA-Na_2_ solution, 0.3 mL riboflavin (200 μmol/L^−1^), 0.1 mL enzyme solution, and 0.2 mL distilled water into a centrifuge tube in turn. After completion of the reaction, the absorbance value was measured at a wavelength of 560 nm. SOD activity was calculated according to the formula. POD activity was determined using the guaiacol method.^36^ Pipette 20 μL of enzyme extract and 3 mL of POD reaction solution into a cuvette, and use the buffer solution used for preparing enzyme solution as blank control to determine the absorbance value at 470 nm wavelength. Read every 30 s, and use the absorbance change value per minute to express the enzyme activity. Determination of CAT activity using visible light colorimetry, pipette 0.1 mL of enzyme extract and 2.9 mL of CAT reaction solution into a cuvette, measure the absorbance value at the wavelength of 240 nm, read it every 10 s, and express the enzyme activity with the absorbance change value per minute.

### 2.6. Determination of reactive oxygen species (ROS)

The production rate of superoxide anion radical (O_2_.^−^) and that content of hydrogen peroxide (H_2_O_2_) were determined by Ingler method.^37,38^ Take 0.5 mL of the supernatant, add 1.5 mL of 65 mM phosphate buffer (pH 7.8) and 0.5 mL of 10 mM hydroxylamine hydrochloride, mix well, and perform a thermostatic water bath at 25°C for 20 min, then add 2 mL of 17 mM sulfanilic acid and 2 mL of 7 mM α-naphthylamine, perform a thermostatic water bath at 30°C for reaction for 30 min, measure the absorbance value at the wavelength of 530 nm, and check the standard curve to obtain the production rate of O_2_.^−^, production rate (nmol min^−1^ g^−1^ FW) = 2 × (NO_2_^−^) × V × N × W^−1^ × T^−1^. V: Volume of reaction solution (mL); N: dilution factor; W: fresh weight of sample (g); T: reaction time (min); NO_2_^−^: NO_2_^−^ concentration (nmol/mL^−1^). Weigh about 0.1 g tissue for ice bath homogenate, take the supernatant and place it on ice for detection, take 200 μL of reaction solution and place it in a 96-well plate, measure the absorbance at 415 nm, and calculate the content of hydrogen peroxide according to the formula.

### 2.7. Determination of malondialdehyde (MDA) content and electrolyte leakage (EL)

According to Bollivar’s description, the content of MDA in foxtail millet leaves was determined.^39^

Weigh 0.5 g leaves, add 2.5 mL PBS buffer solution, grind and homogenize in ice bath, add 2.5 mL PBS, mix well, centrifuge at 10,000 r/min^−1^ for 15 min at 4°C, and the supernatant is crude enzyme extract. Take 1 mL of enzyme extract, add 4 mL of 20% trichloroacetic acid (TCA) solution (containing 0.5% TBA), put it in boiling water for 20 min, cool it with ice water, centrifuge it at 4000 r/min^−1^ for 10 min, take the supernatant to determine the absorbance value at 450 nm, 532 nm and 600 nm, and calculate the MDA content. The relative conductivity method was used to determine the electrolyte leakage of leaves.^21^ Take 10 leaves with equal length and width for each treatment, and three repetitions were conducted for each treatment; Use 10 mL distilled water bubble, put it in a shaker at 28°C for 2 h, measure the conductivity EC1 with a conductivity meter at this time, cool it in a boiling water bath at 100°C for 15 min, and measure the conductivity EC2. Calculation formula: electrolyte permeability (%) = EC1/EC2 × 100%.

### 2.8. Total RNA extraction and qRT-PCR detection

In order to detect the gene expression level, the leaves of foxtail millet seedlings of different treatment groups were put into liquid nitrogen and fully ground, and the total RNA was extracted by RNA extraction kit (Tianmo Biological, used in combination with TRIZOL). RNA concentration was determined by measuring optical density at 260 nm using ultramicro high precision ultraviolet spectrophotometry (ND2000, USA), while RNA integrity was tested by 1% agarose gel electrophoresis. Reverse transcription kit (TaKaRa) was used to obtain cDNA, and the specific operation method was carried out according to the instructions. The PCR products were labeled with TB Green® Premix Ex Taq^TM^ 11 (Tli RNaseH Plus) fluorescence quantitation kit (Takara). The analysis was performed by Applied Biosystems 7500 Fast Real-Time PCR System and StepOnePlus Real-Time PCR System. Primers used for qRT-PCR are listed in Table 1.

**Table 1.** The sequences of genes and their primers used for qRT-PCR.

Gene Name	Forward Primer Sequence (5‘−3’)	Reverse Primer Sequence (5‘−3’)
*SiHAK23*	CCCCCACATCTTCTCCCACT	CTTGTACCCGTACCTCGCCA
*SiRLK35*	AAATAGTTGGGTAGACGAGG	TACGCAGATGATTGACAGAT
*SiASR4*	ACCACCACCACGACAAGAACAAG	CTGCGACGGCACCGACCT

### 2.9. Data statistics and analysis

The experimental data were sorted and calculated by Microsoft Excel 2020 software, and one-way ANOVA was performed by IBM SPSS Statistics 27 software, with *P* <.05 as the standard to test the significance of the difference. Figures were drawn with Origin 64. Each datum is represented as the mean standard deviation of 3 replicates.

## 3. Results

### 3.1. Effects of different concentrations of NaHS on the growth of millet seedlings under salt stress

**Figure 1.**
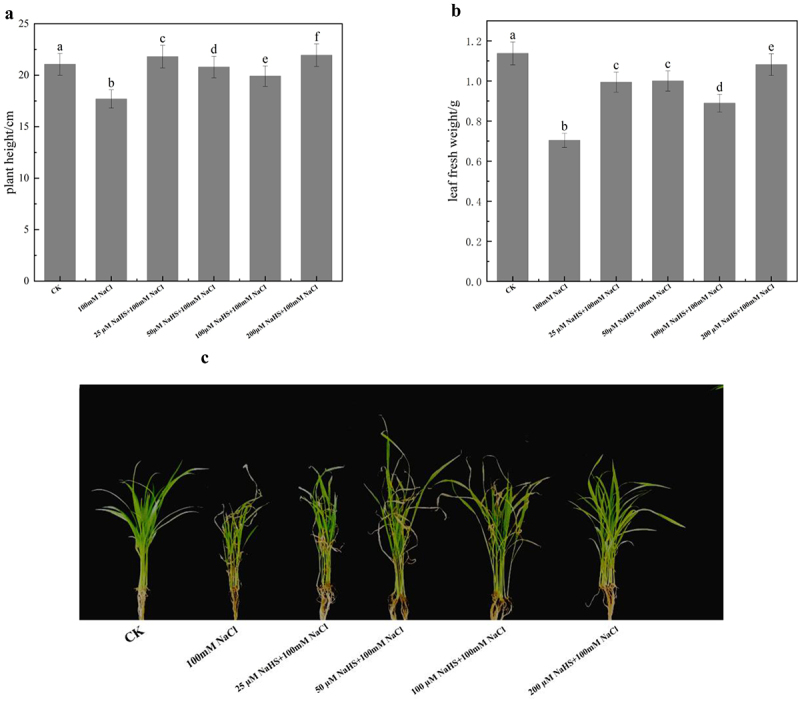
Effects of different concentrations of NaHS on the growth of millet seedlings under salt stress. a: plant height; b: leaf fresh weight, c: growth phenotypes. Each value is the mean of three biological replicates, with different lowercase letters indicating significant differences between treatments (P＜.05). 1-C shows the phenotype of different groups after 7 days of treatments.

Salt stress will affect the carbon assimilation of plants, resulting in reduced plant growth and development. As can be seen from Figure 1a, the application of various concentrations (25, 50, 100, and 200 μM) of sodium hydrosulfide led to a noticeable improvement in plant height compared to the 100 mM NaCl treatment group, demonstrating a mitigating effect of NaHS. Notably, the 200 μM NaHS treatment group exhibited the most remarkable improvement, displaying the highest plant height among all treatment groups. In conclusion, 200 μM NaHS could alleviate the effect of salt stress on plant height of millet seedlings. As shown in Figure 1b, compared with the control, salt stress significantly reduced the fresh weight of millet seedling leaves, indicating that salt stress did harm to the growth of millet seedlings, but the fresh weight of millet seedlings increased under treatment with different NaHS concentrations. As shown in Figure 1b, 200 μM NaHS significantly increased the fresh weight of millet leaves under salt stress. From Figures 1a & 1b, it can be seen that 200 μM NaHS pretreatment can notably increase the plant and leaf fresh weight of millet seedlings under salt stress. Therefore, we selected 200 μM as the final treatment concentration in the subsequent experiments. The growth state of plants is the most intuitive external representation, and plants will show different external forms under different environmental conditions. According to the observation and comparison of Figure 1c, it can be seen that: the millet seedlings in control group were in good growth condition, the leaves were stretched, emerald green, and the leaf margins were smooth and complete; after treatment with 100 mM NaCl, seedlings showed the symptoms of salt stress. The growth quantity and growth rate of millet seedlings decreased, leading to the death of some seedlings. The leaves became shriveled and withered, and dark brown spots and burned leaf tip and edge appeared on the leaves, and there was evidence of deciduous leaves. Compared with the salt stress group, the 200 μM NaHS pretreated group showed a tendency to reduce the salt stress symptoms, the growth volume and growth rate of millet seedlings increased, which led to an alleviation of chlorosis, leaf shrinkage, wilting, and spot symptoms. Thereby, the frequency of leaf drop was significantly reduced.

### 3.2. Effects of NaHS on endogenous hydrogen sulfide content in leaves of millet seedlings under salt stress

**Figure 2.**
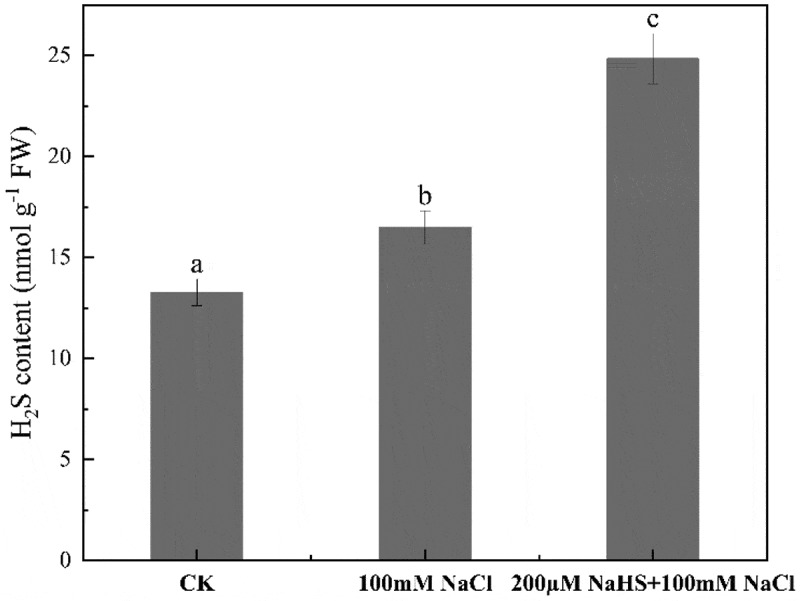
Effect of NaHS on endogenous hydrogen sulfide content of millet seedlings under salt stress. Each value is the mean of three biological replicates, with different lowercase letters indicating significant differences between treatments (P＜.05).

Compared with CK, an advance of endogenous H_2_S content was observed in seedlings under salt stress. Moreover, the results showed that the endogenous H_2_S content in millet seedling leaves increased by 33.7% with pretreatment of 200 μM NaHS compared with the group of 100 mM NaCl. (Figure 2).

### 3.3. Effect of NaHS on chlorophyll content of millet seedling leaves under salt stress

**Figure 3.**
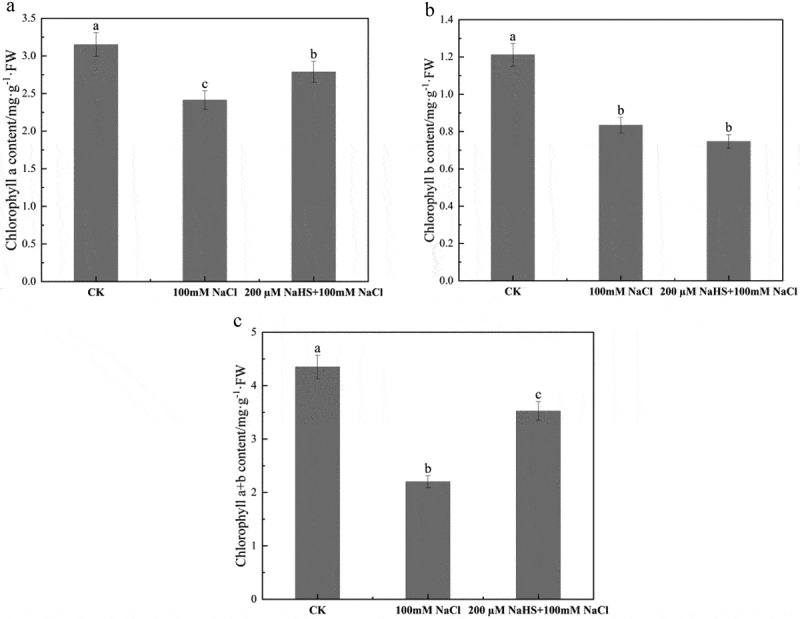
Effects of NaHS on chlorophyll a content and chlorophyll b content of millet seedlings under salt stress. a: chlorophyll a content; b: chlorophyll b content; c: chlorophyll a + b content. Each value is the mean of three biological replicates, with different lowercase letters indicating significant differences between treatments (P＜.05).

As an important photosynthetic pigment, chlorophyll participates in the uptake, transmission, and transformation of light energy in the process of photosynthesis in higher plants. The results can be seen in Figure 3a, the mean concentration of chlorophyll a in the control group (CK) was 3.15 mg/L, while the average chlorophyll a content in the treatment group exposed to 100 mM NaCl was 2.41 mg/L. Notably, in the group treated with 200 µM NaHS +100 mM NaCl, the mean chlorophyll a content was 2.79 mg/L. As can be seen from Figure 3b, the average content of chlorophyll b was 1.21 mg/L, 0.83 mg/L, and 0.75 mg/L in control group, 100 mM NaCl treatment group and 200 µM NaHS +100 mM NaCl treatment group, respectively. Under salt stress, chlorophyll b content of millet seedlings decreased, but NaHS pretreatment did not alleviate the decrease of chlorophyll b content caused by salt stress. As evidenced by Figure 3c, the total chlorophyll content of millet seedling leaves in CK was 4.4 mg/L; Compared with CK, the total chlorophyll content was 2.2 mg/L under 100 mM NaCl treatment, which was significantly decreased by 49.4%; Compared with 100 mM NaCl treatment, the total chlorophyll content of millet seedling leaves increased by 1.6 times under 200 µM NaHS +100 mM NaCl treatment.

### 3.4. Effects of NaHS on leaf membrane damage of millet seedlings under salt stress

**Figure 4.**
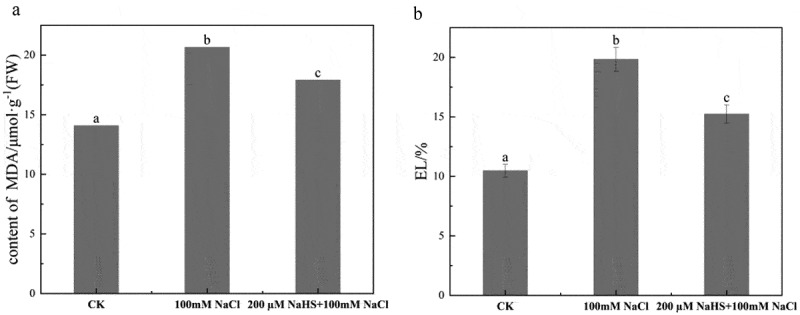
Effect of NaHS on the damage degree of leaf membrane of millet seedlings under salt stress. a: content of MDA; b: EL. Each value is the mean of three biological replicates, with different lowercase letters indicating significant differences between treatments (P＜.05).

In order to study the effects of exogenous H_2_S on MDA and EL in millet seedling leaves under salt stress, MDA content and electrolyte permeability of three groups of millet seedling leaves were determined. Compared with CK, the content of MDA in the leaves of foxtail millet seedlings treated with 100 mM NaCl was significantly higher than that of CK, which was 1.5 times of that of CK. The MDA content of millet seedling leaves treated with 200 μM NaHS +100 mM NaCl increased by 24% compared with CK, and the MDA content of millet seedling leaves treated with 200 μM NaHS +100 mM NaCl decreased significantly by 13.3% compared with 100 mM NaCl group (Figure 4a). Compared with CK, the relative electrical conductivity of foxtail millet seedlings treated with 100 mM NaCl increased by 47%. Compared with the treatment of 100 mM NaCl, the relative electrical conductivity of foxtail millet seedlings treated with 200 μM NaHS +100 mM NaCl decreased significantly by 23% (Figure 4b).

### 3.5. Effects of NaHS on active oxygen species in millet seedlings leaves under salt stress

**Figure 5.**
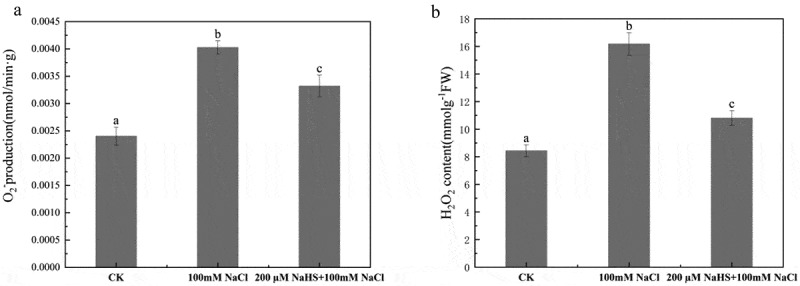
Effects of NaHS on ROS in millet seedlings under salt stress. a: rate of oxygen radical production; b: H_2_O_2_ content. Each value is the mean of three biological replicates, with different lowercase letters indicating significant differences between treatments (P＜.05).

Reactive oxygen species (ROS) are chemically reactive substances with strong oxidative capacity and are natural by-products of the normal metabolism of oxygen. The degree of membrane lipid peroxidation of millet seedlings under salt stress was determined by measuring the production rate of O_2_.^−^ and the content of H_2_O_2_ in the leaves of millet seedlings. Compared with CK, 100 mM NaCl treatment increased the production rate of superoxide anion-free radicals by 40.4%. Compared with 100 mM NaCl treatment, 200 μM NaHS +100 mM NaCl treatment decreased the rate of superoxide anion radical production in millet seedling leaves by 17.6% (Figure 5a). The H_2_O_2_ content in the leaves of foxtail millet seedlings of control group was the lowest (8.4 mmol/g). Compared with CK, the H_2_O_2_ content of 100 mM NaCl treatment was significantly increased to 16.1 mmol/g, which was 1.9 times higher than that of CK. The H_2_O_2_ content of 200 μM NaHS +100 mM NaCl treatment group was higher than CK, but lower than 100 mM NaCl, which was 10.80967 mmol/g, indicating that 200 μM NaHS significantly alleviated the increase of H_2_O_2_ content caused by salt stress (Figure 5b).

### 3.6. Effects of NaHS on antioxidant enzyme activities in millet seedlings leaves under salt stress

**Figure 6.**
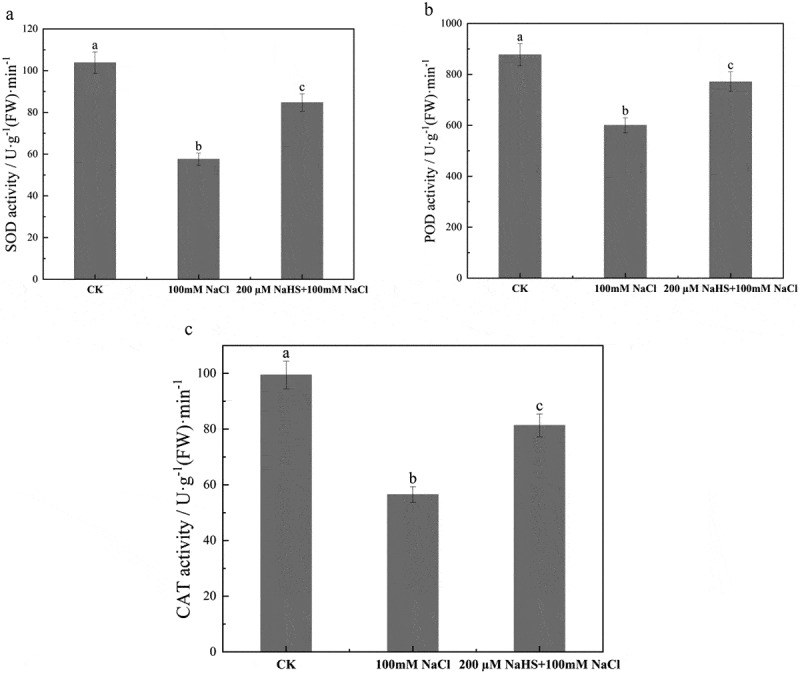
Effect of NaHS on antioxidant enzyme activity of millet seedlings under salt stress. a: SOD activity; b: POD activity; c: CAT activity. Each value is the mean of three biological replicates, with different lowercase letters indicating significant differences between treatments (P＜.05).

The activity of antioxidant enzymes in plants is an effective way to improve plant stress resistance,^40^ and the activities of SOD, POD, and CAT in millet seedlings under three different treatments were determined. As shown in Figure 6a–c, compared with CK, the activities of SOD, POD, and CAT decreased by 44.5%, 18.4%, and 31.6% under 100 mM NaCl treatment, respectively. SOD, POD, and CAT activities of seedlings under 200 μM NaHS +100 mM NaCl treatment increased by 1.5 times, 1.3 times and 1.4 times, respectively, compared with 100 mM NaCl treatment, indicating that 200 μM NaHS could increase SOD, POD, and CAT activities under salt stress (Figure 6a–c).

### 3.7. Effects of NaHS on osmotic adjustment substances in leaves of millet seedlings under salt stress

**Figure 7.**
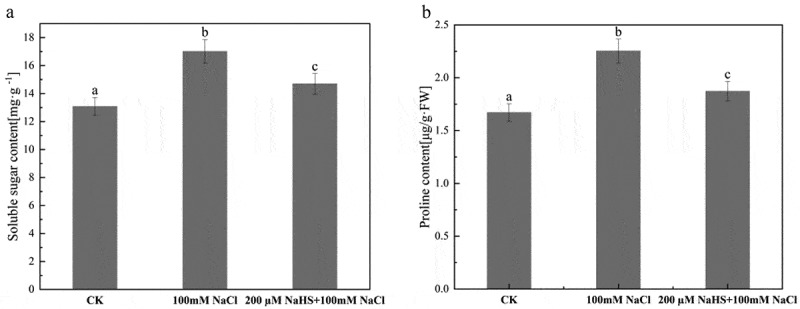
Effect of NaHS on osmotic regulatory substances in millet seedlings leaves under salt stress. a: soluble sugar content; b: proline content. Each value is the mean of three biological replicates, with different lowercase letters indicating significant differences between treatments (P＜.05).

Soluble sugar and proline content are important indexes for plants to resist abiotic stress. According to Figure 7a, compared with CK, 100 mM NaCl treatment decreased the soluble sugar content in the leaves of millet seedlings, and the soluble sugar content in the leaves of millet seedlings decreased significantly after spraying 200 μM NaHS. It can be seen from Figure 7b that compared with CK, both of the Pro content of millet seedling leaves treated with 100 mM NaCl and 200 μM NaHS +100 mM NaCl were significantly increased. The Pro content of millet seedling leaves treated with 100 mM NaCl was significantly lower than that of 200 μM NaHS +100 mM NaCl millet seedling leaves.

### 3.8. Effects of NaHS on the expression of related genes in leaves of millet seedlings under salt stress

To investigate the involvement of related genes in alleviating salt stress, we conducted real-time quantitative PCR verification on *SiASR4*, *SiRPLK35*, and *SiHAK23*. We measured the gene expression changes of *SiASR4*, *SiRPLK35*, and *SiHAK23* in millet under different treatment conditions. The results demonstrated that under 100 mM NaCl treatment and 200 μM NaHS +100 mM NaCl treatment, the expression levels of *SiASR4* and *SiRPLK35* genes in foxtail millet seedling leaves increased by 39.7%, 59.7%, 12.8%, and 53.4%, respectively, compared to CK. Furthermore, compared to CK, the expression level of the *SiHAK23* gene in millet seedling leaves decreased by 19.1% under 100 mM NaCl treatment and increased by 41.4% under 200 μM NaHS +100 mM NaCl treatment.

## 4. Discussion

Studies have found that the plants growth and physiology were significantly affected by the content of salt ions in soil, and excessive salt content will disturb the metabolism of plants.^41^ Therefore, the study of the impact of salt stress on plant phenotype has always been one of the most basic research fields of plant stress physiology. The research of Mukami et al. showed that under the condition of salt stress, the growth of plants was severely inhibited, resulting in significant changes in the morphology and structure of the seedling height, leaf, and root length, which was manifested as a slowdown in the growth rate of seedling height, shrinkage, and yellowing of the leaves.^42^ In severe cases, the leaves will wilt and wither, and the growth of the root system will be hindered, which may even lead to premature aging or death of the plant. The researches of Haider et al. and Liu et al. showed that under the condition of salt stress, the growth physiological indicators of plants such as growth height, leaf number, root biomass, root-shoot ratio, and aboveground biomass all showed a downward trend.^43,44^ In this study, millet seedlings in 100 mM NaCl solution treatment exhibited reduced growth, resulting in plant stunting and the occurrence of withered leaves (Figure 1). The pretreatment of 200 μM NaHS increased the plant height of millet seedlings under salt stress. Exogenously applied NaHS effectively alleviated the damage of salt stress on millet photosynthetic system of millet, leading to increased chlorophyll content in leaves and enhanced accumulation of dry matter. Consequently, the plant height and fresh weight of millet seedlings subjected to salt stress were significantly augmented (Figure 1).

As an important photosynthetic pigment, chlorophyll participates in the uptake, transmission and conversion of light energy in the photosynthesis process of higher plants, and the strength of plant photosynthesis is closely related to the content of chlorophyll.^45^ In this study, it can be seen from Figure 3 that the content of chlorophyll a and chlorophyll b in millet seedlings decreased under salt stress, which is consistent with the research results of Zai et al.^46^ In our results, the content of chlorophyll a in seedlings sprayed with 200 μM NaHS increased by 15.77% compared with salt-treatment group, while the content of chlorophyll b in seedlings sprayed with 200 μM NaHS decreased by 9.64% compared with salt-treatment group. Nevertheless, our analysis of the total chlorophyll content, comprising chlorophyll a and chlorophyll b, unequivocally demonstrated that NaHS pretreatment significantly enhanced the overall chlorophyll levels in millet seedlings under salt stress (Figure 3), indicating that 200 μM NaHS could inhibit the reduction of chlorophyll under salt stress.

Raju’s et al. research showed that NaHS significantly alleviated the effect of salt stress on the relative conductivity of sweet-scented clover seedling leaves, and exogenous H_2_S mitigated the effects of salt stress on plants by reducing the permeability of cell membranes damage.^38^ The cell membrane is the interface and barrier between living cells and the environment. The impact of various adverse environments on cells often acts on the cell membrane first, MDA is the product of membrane lipid peroxidation. Salt stress leads to excessive accumulation of MDA, leading to the damage of cell membrane system and increment of electrolyte permeability.^47,48^ Generally, the more serious the damage to plants, the greater the relative conductivity of leaves, otherwise the smaller it is. Research has proved that the MDA content of tomato seedlings treated with NaHS decreased under salt stress.^49^ NaHS also has a significant effect on the decrease of malondialdehyde in naked oat leaves under salt stress.^46^ In this study, the content of malondialdehyde in the leaves of millet seedlings significantly increased by NaCl solution treatment, but decreased with applying 200 μM NaHS, which proved that 200 μM NaHS could alleviate the damage caused by salt stress to the leaves of millet seedlings. After NaCl solution treatment, the cellular water content of millet seedling leaves was depleted, resulting in the disruption of cell membrane structure and increased membrane permeability. But the value decreased in 200 μM NaHS pretreatment group, which indicated that 200 μM NaHS could mitigate the deleterious effects caused by salt stress on millet seedling leaves (Figure 4).

As reactive oxygen species, O_2_.^−^and H_2_O_2_ can participate in regulating the response of plants to adversity stress, but excessive accumulation of ROS will damage biological macromolecules and cause oxidative damage to plant cell plasma membrane.^50^ Zhao et al. showed that salt stress would lead to the accumulation of reactive oxygen species in plants.^51^ Raju et al. studied the effect of NaHS on tomato growth physiology under salt stress and found that O_2_.^−^ and H_2_O_2_ content increased significantly under NaCl stress, while decreased dramatically under NaHS pretreatment.^38^ Results showed that the accumulation of O_2_.^−^ and H_2_O_2_ in rice seedling leaves increased under the treatment of 100 mM NaCl, while exogenous application of H_2_S could significantly inhibit the accumulation of O_2_.^−^and H_2_O_2_ in leaves induced by salt stress.^52^ In this study, as shown in Figure 5b, compared with CK group, the production rate of superoxide anion radical in seedling leaves was greatly increased under salt stress, and markedly reduced after exogenous use of hydrogen sulfide, which was consistent with previous papers.^52^ As evident from Figure 5b, H_2_O_2_ content of millet seedlings increased noticeably under salt stress, and a considerable decline was observed in 200 μM NaHS pretreatment group, which was consistent with the research of A Raza et al.^53^ This indicates that the oxidative damage was occurred in seedlings under salt stress, and the toxicity caused by salt stress could be weakened by the positive function of NaHS.

The activity of antioxidant enzymes in plants indicates the degree of oxidative damage, which is an important index to study the response of plants to abiotic stress.^52^ When plants are exposed to a high-salt environment, the dynamic balance between generation and consumption of ROS in plants will be broken, which will lead to a reduced activity of antioxidant enzymes, resulting in serious oxidative damage to plants.^54^ The research of Liang et al showed that when salt stress intensified, the activity of antioxidant enzymes in plant leaves decreased and the membrane lipid peroxidation strengthened, resulting in plant damage.^54^ There are also studies on rice seedlings under the condition of salt stress,^55^ the activities of POD and CAT in the seedlings decreased obviously. In this study, the activities of SOD, POD, and CAT in leaves of millet seedlings were all decreased after being treated with 100 mM NaCl, on the contrary, all of them raised in 200 μM NaHS +100 mM NaCl group compared with separate salt treatment group. The results proved that 200 μM NaHS treatment could effectively alleviate the oxidative damage to millet seedlings caused by salt stress and reduce the accumulation of reactive oxygen species through enhancing the activities of antioxidant enzymes (Figure 6).

The accumulation of free proline and soluble sugar in plants is one of the important mechanisms to resist abiotic stress.^56^ In the study of salt stress-induced accumulation of soluble sugar content in eggplant, it was pointed out that salt stress would induce the accumulation of soluble sugar content in eggplant, and spraying H_2_S on leaves reduced the accumulation of soluble sugar under salt stress,^50^ which were consistent with our results (Figure 7a). Research showed that proline content of wheat seedlings was greatly increased by high temperature and drought stress, but the increase of proline content was reduced by NaHS treatment, which indicated that H_2_S played a positive role in reducing the harm of environmental stress to wheat seedlings.^57^ Compared with the control group, the proline content in the leaves of millet seedlings treated with 100 mM NaCl increased substantially, while the proline content in the millet pretreated with 200 μM NaHS decreased after salt stress (Figure 7b), and the damage degree was reduced, which was basically consistent with the experimental results of Wu et al.^57^

**Figure 8.**
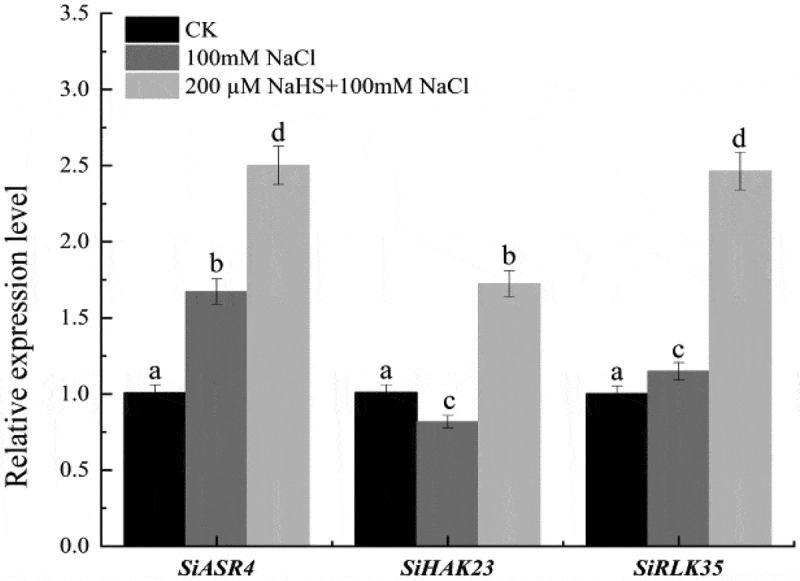
Effects of NaHS on the expression of genes *SiASR4*, *SiHAK23* and *SiRPLK35* in leaves of millet seedlings under salt stress. Each value is the mean of three biological replicates, with different lowercase letters indicating significant differences between treatments (P＜.05).

**Figure 9.**
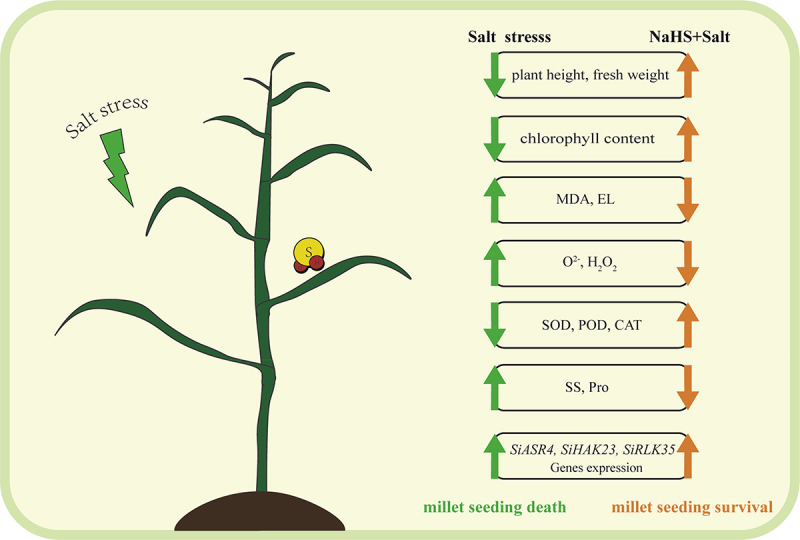
The model of NaHS alleviating salt stress in millet seedlings. The model revealed how NaHS mitigated the damage of millet seedlings under salt stress. Under salt stress, on the one hand, NaHS pretreatment in advance increased the salt tolerance of millet seedlings by up-regulating the expression of *SiASR4*, *SiHAK23*, and *SiRPLK35* genes, on the other hand, NaHS pretreatment significantly increased the activities of antioxidant enzymes SOD, POD and CAT, and decreased the content of ROS. In this model, the green arrows represent the trend of indicator changes in the salt-treated group compared to the control. The orange arrows represent the trend of indicator changes in the NaHS pre-treated group compared to the salt-treated group.

Under salt stress, Na^+^ was markedly reduced and proline content was significantly increased in transgenic tobacco compared to wild-type tobacco, indicating that tomato ASR1 plays an important role in osmotic and ionic stress.^58^ *SiASR4* in foxtail millet was proved to be involved in plant responses to abiotic stress through ABA signaling pathway, and played an important role in plant drought resistance and salt stress. The results showed that the heterologous expression of *SiASR4* in *Arabidopsis thaliana* could improve the tolerance of transgenic plants to drought and salt stress, and the accumulation of reactive oxygen species in transgenic *Arabidopsis thaliana* was reduced under drought and salt stress. In *Arabidopsis thaliana* and rice, the expression of *HAKs* was significantly increased after salt stress, which may be involved in maintaining K^+^/Na^+^ balance in plants to cope with high salt stress.^59^ The expression levels of *SiHAK23* and *SiHAK3* in foxtail millet under high salt stress were about 9 times and about 4 times higher than those in the control (Figure 8). The change of *SiHAKs* gene expression level indicated that some *SiHAKs* may be involved in the K^+^ uptake process in response to salt stress.^40,56^ *LecRLKs* (Lectin receptor-like protein kinases) are the largest gene family in plants and play an important role in regulating plant growth and development, stress resistance and disease resistance.^60^ A large number of studies have shown that *LecRLKs* expression is significantly up-regulated under salt stress conditions, which can reduce the accumulation of ROS to improve the salt tolerance of plants. *PnRLK-1* is a cytoplasmic receptor-like protein kinase in *Antarctic mosses*. Under salt treatment, *PnRLK-1* can up-regulate the expression of a series of ROS scavenging genes such as *AtAPX1*, *AtZAT10*, and *AtCAT1*, thereby reducing ROS accumulation and improving plant tolerance to salt stress.^60^ In this study, the expression levels of *SiASR4*, *SiRPLK35*, and *SiHAK23* genes in millet seedling leaves under salt stress were significantly increased after exogenous application of NaHS. We hypothesized that NaHS pretreatment could up-regulate the expression levels of *SiASR4*, *SiHAK23*, and *SiRPLK35* genes in the leaves of foxtail millet seedlings, and then activate a series of antioxidant enzyme activities to reduce the accumulation of reactive oxygen species in seedlings, while maintaining the balance of K^+^/Na^+^ in plants, and finally improve the ability of foxtail millet seedlings to resist salt stress and enhance their salt tolerance (Figure 9).

## 5. Conclusion

Our research showed that salt stress had significant adverse effects on the growth and physiology of millet seedlings. Exogenous application of 200 μM NaHS in advance could activate the expression levels of *SiASR4*, *SiRPLK35*, and *SiHAK23*, improve the growth and photosynthetic capacity of millet seedlings under salt stress, increase the activity of antioxidant enzymes, reduce the accumulation of reactive oxygen species, and actively regulate osmotic substances, thus enhancing the salt tolerance of millet under NaCl stress. The results showed that 200 μM NaHS could significantly alleviate the toxicity of NaCl stress on millet seedlings. This study provided a theoretical framework for the cultivation of salt-tolerant millet seedlings. We believe that it holds significant guidance for the scientific utilization of saline-alkali soil, enhancing grain yield, and improving the ecological environment.

## References

[1] Yang Y, Cheng J, Han H, Sun R, Li Y, Zhang Y, Han Y, Zhang H, Li X. Genome-wide identification of the HKT transcription factor family and their response to salt stress in foxtail millet (setaria italica). Plant Growth Regul. 2023;99(1):113–12. doi:10.1007/s10725-022-00903-z.

[2] Abah CR, Ishiwu CN, Obiegbuna JE, Oladejo AA. Nutritional composition, functional properties and food applications of Millet Grains. Asian Food Sci J. 2020;14(2):9–19. doi:10.9734/afsj/2020/v14i230124.

[3] Meng L, Yu Z, Shun Guo L. Current situation and development suggestion of foxtail millet production in the Taihang Mountain, Hebei Province——taking foxtail millet production investigation in Wu’an city as a case. J Agric. 2011;6(10):e26550–e26550. doi:10.1371/journal.pone.0026550.

[4] Jian-Bing LI, Shou-Guo H, Cai-Ping W. Thinking on the production and Industry development of millet in Shanxi Province. J Shanxi Agric Sci. 2015;43(11):1508–1510. in Chinese.

[5] Shun-Guo LI, Fei L, Meng L. The current industry situation, development trend, and suggestions for the future of foxtail millet in China. Res Agric Modernization. 2014;35(5):531–535. in Chinese.

[6] Radi AA, Farghaly FA, Hamada AM. Physiological and biochemical responses of salt-tolerant and salt-sensitive wheat and bean cultivars to salinity. J Biol Earth Sci. 2013;3:72–88.

[7] Li H, Shi J, Wang Z, Zhang W, Yang H. H_2_S pretreatment mitigates the alkaline salt stress on Malus hupehensis roots by regulating Na^+^/K^+^ homeostasis and oxidative stress. Plant Physiol Bioch. 2020;156:233–241. doi:10.1016/j.plaphy.2020.09.009. 32977178

[8] Gou QQ, Han ZW, Wang GH. Research progress on soil salinization in arid irrigated area of Northwestern China. Chinese Agric Sci Bull. 2011;27(29):246–250. in Chinese.

[9] Jafar MZ, Farooq M, Cheema, M A, Afzal I, Basra SMA, Wahid MA, Aziz T, Shahid M. Improving the performance of wheat by seed priming under saline conditions. J Agron Crop Sci. 2012;198(1):38–45. doi:10.1111/j.1439-037X.2011.00485.x.

[10] Raza A, Tabassum J, Fakhar AZ, Sharif R, Chen H, Zhang C, Ju L, Fotopoulos V, Siddique KHM, Singh RK, et al. Smart reprograming of plants against salinity stress using modern biotechnological tools. Critical Reviews In Biotechnology. 2022;43(7):1035–1062. doi:10.1080/07388551.2022.2093695. 35968922

[11] Ustinov M, Glistin M. Geosystem evaluation of genetic and meliorative peculiarities of soils ofsodal salinization. Melioration Water Manage. 2020;10(12):31–34. doi:10.32962/0235-2524-2019-4-31-34.

[12] Zhou WH, Feng RZ, Shi SL, Kou J. Nitric oxide protection of alfalfa seedling roots against salt-induced inhibition of growth and oxidative damage. Acta Ecologica Sinica. 2015;35(11):3606–3614. doi:10.5846/stxb201310142472.

[13] Correa-Aragunde N, Graziano M, Lorenzo L. Nitric oxide plays a central role in determining lateral root development in tomato. Planta. 2004;218(6):900–905. doi:10.1007/s00425-003-1172-7. 14716561

[14] Huang YR, Lin WX, Nie S, Zhu W. Changes of antioxidant metabolism and organic solute accumulation of Pittosporum pentan-drum and Koelreuteria elegans seedlings under salt stress. Chinese J Ecol. 2014;33:3176–3183.

[15] Yu CG, Li Y, Xie YF, Yin YL. Effects of NaCl stress on growth and absorption, transportation and distribution of ions in zhongshanshan seedlings. Plant Physiol J. 2016;52(9):1379–1388. doi:10.13592/j.cnki.ppj.2016.0272.

[16] Chinnusamy V, Jagendorf A, Zhu JK. Understanding and improving salt tolerance in plants. Crop Sci. 2005;45(2):437–48. doi:10.2135/cropsci2005.0437.

[17] Xiang BY, Qing LI, Shao Wu W. Research progress of exogenous substances for alleviate seed germination under salt stress. Heilongjiang Agric Sci. 2013; 11:147.

[18] Hosoki R, Matsuki N, Kimura H. The possible role of Hydrogen sulfide as an endogenous smooth muscle relaxant in synergy with Nitric oxide. Biochem Bioph Res Co. 1997;237(3):527–531. doi:10.1006/bbrc.1997.6878. 9299397

[19] Zhang H, Tang J, Liu X-P, Wang Y, Yu W, Peng W-Y, Fang F, Ma D-F, Wei Z-J, Hu L-Y. Hydrogen sulfide promotes root organogenesis in Ipomoea batatas, Salix matsudana and Glycine max. JIPB. 2009;51(12):1086–1094. doi:10.1111/j.1744-7909.2009.00885.x. 20021556

[20] Wang LJ, Mu XJ, Chen X, Han Y. Hydrogen sulfide attenuates intracellular oxidative stress via repressing gly‐ colate oxidase activities in Arabidopsis thaliana. BMC Plant Biol. 2022;22(1):98. doi:10.1186/s12870-022-03490-3. 35247968 PMC8897949

[21] Yoo CY, Pence HE, Jin JB, Miura K, Gosney MJ, Hasegawa PM, Mickelbart MV. The Arabidopsis GTL1 transcription factor regulates water use efficiency and drought tolerance by modulating stomatal density via transrepression of SDD1. The Plant Cell. 2010;22(12):4128–4141. doi:10.1105/tpc.110.078691. 21169508 PMC3027182

[22] Turan M, Ekinci M, Kul R, Boynueyri FG, Yildirim E. Mitigation of salinity stress in cucumber seedlings by NaHS. J Plant Res. 2022;135(3):517–529. doi:10.1007/s10265-022-01391-y. 35445911

[23] Fang HH, Jing T, Liu ZQ, Zhang L, Jin Z, Pei Y. Hydrogen sulfide interacts with calcium signaling to enhance the chromium tolerance in Setaria italica. Cell Calcium. 2014;56(6):472–481. doi:10.1016/j.ceca.2014.10.004. 25459298

[24] Mostofa MG, Daisuke S, Masayuki F, Tran LSP. Hydrogen sulfide regulates salt tolerance in rice by maintaining Na^+^/K^+^ balance, mineral homeostasis and oxidative metabolism under excessive salt stress. Front Plant Sci. 2015;6:1055. doi:10.3389/fpls.2015.01055. 26734015 PMC4685665

[25] Henry IM, Carpentier SC, Pampurova S, Van Hoylandt A, Panis B, Swennen R, Remy S. Structure and regulation of the asr gene family in banana. Planta Int J Plant Biol. 2011;234(4):785-798.234785–798. doi:10.1007/s00425-011-1421-0. PMC3180632 21630042

[26] Kalifa Y, Gilad A, Konrad Z, Zaccai M, Scolnik P, Bar-zvi D. The water- and salt-stress-regulated *Asr1* (abscisic acid stress ripening) gene encodes a zinc-dependent DNA-binding protein. Biochem J. 2004;381(2):373–378. doi:10.1042/bj20031800. 15101820 PMC1133842

[27] Kalifa Y, Perlson E, Gilad A, Konrad Z, Scolnik PA, Bar‐zvi D. Over‐expression of the water and salt stress‐regulated *Asr1* gene confers an increased salt tolerance. Blackwell Sci Ltd. 2004;27(12):1459–1468. doi:10.1111/J.1365-3040.2004.01251.X.

[28] Li W, Xu G, Alli A, Yu L. Plant HAK/KUP/KT K ^+^ transporters: function and regulation. Semin Cell Dev Biol. 2017;74:S1084952116303688. doi:10.1016/j.semcdb.2017.07.009. 28711523

[29] Gierth M, Mäser P. Potassium transporters in plants – involvement in K + acquisition, redistribution and homeostasis. FEBS Lett. 2007;581(12):2348–2356. doi:10.1016/j.febslet.2007.03.035. 17397836

[30] Vaid N, Pandey P, Srivastava VK, Tuteja N. Pea lectin receptor-like kinase functions in salinity adaptation without yield penalty, by alleviating osmotic and ionic stresses and upregulating stress-responsive genes. Plant Mol Biol. 2015;88(1–2):193–206. doi:10.1007/s11103-015-0319-9. 25863480

[31] Yifan W, Zhen L, Jiaowen P, Li Y, Wang Q, Guan YA, Liu W. Cloning and functional analysis of the *SiRLK35* gene in *Setaria italic L*. Yi Chuan = Hereditas. 2017;39(5):413. doi:10.16288/j.yczz.17-027. 28487273

[32] Jianrui L, Yang D, Cong L, Pan Y, Yu J. SiASR4, the target gene of SiARDP from *Setaria italica*, improves abiotic stress adaption in plants. Front Plant Sci. 2016;7:2053. doi:10.3389/fpls.2016.02053. 28127300 PMC5227095

[33] Qiao ZJ, Jing T, Jin ZP, Liang YL, Zhang LP, Liu ZQ, Liu D, Pei Y. Cdpks enhance cd tolerance through intensifying H_2_S signal in Arabidopsis thaliana. Plant Soil. 2016;398(1–2):99–110. doi:10.1007/s11104-015-2643-x.

[34] Bates LS, Waldren RP, Teare ID. Rapid determination of free proline for water-stress studies. Plant Soil. 1973;39(1):205–207. doi:10.1007/BF00018060.

[35] Shams M, Ekinci M, Ors S, Turan M, Agar G, Kul R, Yildirim E. Nitric oxide mitigates salt stress effects of pepper seedlings by altering nutrient uptake, enzyme activity and osmolyte accumulation. Physiol Mol Biol Plants: Int J Funct Plant Biol. 2019;25(5):1149–1161. doi:10.1007/s12298-019-00692-2. PMC6745581 31564778

[36] Chen T, Zhang B. Measurements of proline and malondialdehyde contents and antioxidant enzyme activities in leaves of drought stressed cotton. BIO-PROTOCOL. 2016;6(17). doi:10.21769/BioProtoc.1913. PMC9999191 36909294

[37] Yin L, Wang S, Tanaka K, Fujihara S, Itai A, Den X, Zhang S. Silicon-mediated changes in polyamines participate in silicon-induced salt tolerance in Sorghum bicolor L. Plant Cell & Environment. 2016;39(2):245–258. doi:10.1111/pce.12521. 25753986

[38] Raju AD, Prasad SM. Hydrogen sulfide implications on easing NaCl induced toxicity in eggplant and tomato seedlings. Plant Physiol Bioch. 2021;164(1):173–184. doi:10.1016/j.plaphy.2021.05.001. 33993067

[39] Bollivar DW. Recent advances in chlorophyll biosynthesis. Photosynth Res: Int J. 2006;90(2):173–194. doi:10.1007/PL00022068. 17370354

[40] Mukami A, Ng’etich A, Syombua E, Oduor R, Mbinda W. Varietal differences in physiological and biochemical responses to salinity stress in six finger millet plants. Physiol Mol Biol Plants. 2020;26(8):1569–1582. doi:10.1007/s12298-020-00853-8. 32801487 PMC7415052

[41] Haider SI, Kang W, Zhang GP. Synergistic interaction of NaCl and cd on growth and photosynthetic parameters in soybean genotypes differing in salinity tolerance. J Zhejiang Univ (Sci B: Int Biomed & Biotechnol J). 2007;8(4):6. doi:10.1631/jzus.2007.B0266. PMC1838833 17444602

[42] Liu B, Kang C, Wang X, Bao G. Physiological and morphological responses of leymus chinensis to saline-alkali stress. Grassl Sci. 2015;61(4):217–226. doi:10.1111/grs.12099.

[43] Zai XM, Zhu SN, Qin P, Wang XY, Che L, Luo FX. Effect of glomus mosseae on chlorophyll content, chlorophyll fluorescence parameters, and chloroplast ultrastructure of beach plum (prunus maritima) under NaCl stress. Photosynthetica. 2012;50(3):323–328. doi:10.1007/s11099-012-0035-5.

[44] Zhou Yuan Z, Hairong L, Huimei C. Effect of NaHS on the physiological and biochemical characteristics of Processing tomato seedlings under NaCl stress. Acta Agricu Boreali-Sinica. 2017.

[45] Zhong Y-H, Guo Z-J, Wei M-Y, Wang J-C, Song S-W, Chi B-J, Zhang Y-C, Liu J-W, Li J, Zhu X-Y, et al. Hydrogen sulfide upregulates the alternative respiratory pathway in mangrove plant Avicennia marina to attenuate waterlogging-induced oxidative stress and mitochondrial damage in a calcium-dependent manner. Plant Cell & Environment. 2023;46(5):1521–1539. doi:10.1111/PCE.14546. 36658747

[46] Mittler R. Oxidative stress, antioxidants and stress tolerance. Trends In Plant Science. 2002;7(9):405–410. doi:10.1016/S1360-1385(02)02312-9. 12234732

[47] Xiao-Jing HU. Influence of low temperature stress on electrolyte permeability and content of MDA of *hippophae rhamnoides* L. J Anhui Agri Sci. 2008;30(8):1008–14. doi:10.3724/SP.J.1005.2008.01008.

[48] Jun L. Effects of infection with botryosphaeria dothidea on Cell membrane permeability, soluble sugar and MDA content in Poplar Calli. Scientia Silvae Sinicae. 2008;44(8):72–77. doi:10.1016/S1872-2040(08)60061-4.

[49] Zhao S, Zhang Q, Liu M, Zhou H, Ma C, Wang P. Regulation of plant responses to salt stress. Int J Mol Sci. 2021;22(9). doi:10.3390/ijms22094609. PMC8125386 33924753

[50] Raza A, Tabassum J, Mubarik MS, Anwar S, Zahra N, Sharif Y, Hafeez MB, Zhang C, Corpas FJ, Chen H, et al. Hydrogen sulfide: an emerging component against abiotic stress in plants. Plant Biol (Stuttgart,germany). 2022;24(4):540–558. doi:10.1111/plb.13368. 34870354

[51] Wei M-Y, Liu J-Y, Li H, Hu W-J, Shen Z-J, Qiao F, Zhu C-Q, Chen J, Liu X, Zheng H-L. Proteomic analysis reveals the protective role of exogenous hydrogen sulfide against salt stress in rice seedlings. Nitric Oxide (Prepublish). 2021;111-112:14–30. doi:10.1016/j.niox.2021.04.002. 33839259

[52] Egbichi I, Keyster M, Jacobs A, Klein A, Ludidi N. Modulation of antioxidant enzyme activities and metabolites ratios by nitric oxide in short-term salt stressed soybean root nodules. S Afr J Bot. 2013;88:326–333. doi:10.1016/j.sajb.2013.08.008.

[53] Yan HE. Responses of the growth and the protective enzymes activities in two genotypic wheats under salt stress. J Shanxi Agric Univ. 2005;25(1):42–4.

[54] Liang Jie X, Jiayue W, Lixin W. Prospect of per capita grain demand driven by dietary structure change in China. Resour Sci. 2015;37(7):1347–1356. in Chinese.

[55] Li W, Xu G, Alli A, Yu L. Plant HAK/KUP/KT K+ transporters: function and regulation. Semin Cell Dev Biol. 2018;74:133–141. doi:10.1016/j.semcdb.2017.07.009. 28711523

[56] Meiyun Z, Qian JI, Shi Zhang Z. Studies on free proline and soluble sugar of wild soybeans (glycine soja)Under osmotic stress. J Fudan Univ. 2001;40(5):558–561. in Chinese.

[57] Wu DH, Li YL, Xun X, Pu ZP, Liao JM, Huang K, Li ZG. Hydrogen sulfide donor sodium hydrosulfide pretreatment improved multiple resistance abilities of wheat to high temperature and drought stress. J Yunnan Normal Univ (Nat Sci Ed). 2013;33:29–35. doi:10.1016/j.semcdb.2017.07.009.

[58] Maathuis F. The role of monovalent cation transporters in plant responses to salinity. J Exp Bot. 2006;57:1137–1147. doi:10.1093/jxb/erj001. 16263900

[59] YeY Y, Ding YF, Jiang Q, Wang FJ, Sun JW, Zhu C. The role of receptor-like protein kinases (RLKs)in abiotic stress response in plants. Plant Cell Report. 2017;36(2):235–242. doi:10.1007/s00299-016-2084-x. 27933379

[60] Zhang PY, Zhang ZH, Wang J, Cong BL, Chen KS, Liu SH. A novel receptor-like kinase (PnRLK-1) from the Antarctic Moss Pohlia nutans enhances salt and oxidative stress tolerance. Plant Mol Biol Rep. 2014;33(4):1156–1170. doi:10.1007/s11105-014-0823-0.

